# Huangbai Liniment Accelerated Wound Healing by Activating Nrf2 Signaling in Diabetes

**DOI:** 10.1155/2020/4951820

**Published:** 2020-05-23

**Authors:** Jingjing Zhang, Rui Zhou, Changpei Xiang, Qiang Jia, Hongwei Wu, Hongjun Yang

**Affiliations:** ^1^Institute of Chinese Materia Medica, China Academy of Chinese Medical Sciences, Beijing 100700, China; ^2^College of traditional Chinese Medicine, Yunnan University of Traditional Chinese Medicine, Kunming 650500, China; ^3^School of Pharmacy, Shandong University of Traditional Chinese Medicine, Jinan 250355, China

## Abstract

As a serious complication of diabetes, nonhealing skin ulcer leads to high mortality and disability in diabetic patients. However, limited therapy is available in managing diabetic wounds. In this study, RNA-seq technology was used to systematically investigate the effect of Huangbai (HB) liniment, a traditional Chinese medicine, on the streptozotocin- (STZ-) induced diabetic wound. HB liniment significantly accelerated the wound closure and enhanced the generation of extracellular matrix in diabetic rats, and oxidative stress was identified to play a vital role in HB-mediated wound healing. Importantly, HB liniment activated nuclear factor erythroid-derived 2-like 2 (Nrf2) and its downstream antioxidant genes (e.g., genes involved in glutathione system, thioredoxin system, and GAPDH generation as well as other antioxidant genes), which inhibited oxidative damage and apoptosis. By associating drug targets of HB liniment with Nrf2 and its downstream genes, 54 components in HB liniment were screened out, and the majority was from Cortex Phellodendri and Forsythia suspensa. Additionally, HB liniment enhanced TGF-*β*1 and reduced MMP9 level, accelerating wound healing in diabetes. The *in vitro* experiment showed HB facilitated cell proliferation and inhibited oxidative damage in high glucose-induced HaCaT cells. Our findings provided the experimental evidence for the treatment of diabetic wound with HB, clarified the potential mechanism of HB, and improved our understanding of diabetic wound healing.

## 1. Introduction

Chronic nonhealing wound is a serious diabetic complication, which leads to severe morbidity and mortality in diabetic population and brings a huge social and economic burden to the word [1, 2]. In the United States alone, it costs as high as $13 billion to treat diabetic wounds every year. In contrast to the typical sequential emergence of biological process of coagulation, inflammation, proliferation, and remodeling in normal tissue, the normal progression of wound healing is disturbed and delayed in diabetes, resulting in long-term of wound nonunion [3, 4]. Conventional therapies are only effective in the management of diabetic wounds to certain degree, whereas a large number of diabetic wounds still persist, deteriorate, and result in amputation [5]. Notably, a reduced efficiency in diabetic wound healing is usually accompanied with decreased blood supply, delayed extracellular matrix turnover, reduced wound contraction, repeated infections, and chronic inflammation, which hinders wound healing [4, 6]. Thus, it is urgent to develop an effective therapy to treat diabetic wounds.

Oxidative stress plays a vital role in halting the progression of diabetic wound healing [7, 8]. The oxidative stress in diabetic wounds is characterized by a marked elevation in reactive oxygen species (ROS) levels, as a result of increasing ROS generating pathway and decreasing ROS removing defenses [9]. Overproduced ROS in diabetes damages various macromolecules such as lipids, proteins, and DNA double strands and finally impairs wound healing. Proper oxidative stress has been proved to benefit extracellular matrix (ECM) generation, whereas high ROS levels hinder normal synthesis of ECM and seriously damage existing ECM [10, 11]. For example, H_2_O_2_ can disrupt tissue growth, especially collagen production, thus preventing wound closure [12]. In addition, TGF-*β*1 signaling is a vital signaling pathway for ECM production and can be interfered by the overproduced ROS. Importantly, high ROS levels promote the generation of matrix metalloproteinases (MMPs), which in turn affect ECM remodeling [13]. Therefore, targeting oxidative stress may be a potential effective therapy for diabetic wound healing.

An increasing number of researchers have demonstrated Traditional Chinese medicine (TCM) is indispensable in the treatment of various disease presentations [14, 15]. Abundant and robust evidences accumulated over time that TCM is effective in its use for diabetic complications [16]. Huangbai liniment (HB) is a standardized medicinal product clinically prescribed for use in wound management and is composed of Cortex Phellodendri, Forsythia suspensa, Lonicera japonica Thunb., Taraxacum mongolicum Hand.-Mazz, and Scolopendridae [17]. Therapeutic benefits of HB liniment have been observed in wound management, including nonhealing diabetic ulcer [18, 19]. These study demonstrated that HB liniment could reduce AGEs and inflammatory factors such as IL-1*β* while increasing the secretion of growth factor [20]. However, the mechanism of HB in facilitating diabetic wound healing remains unclear. In this study, the effect of HB on diabetic wound healing was evaluated in STZ-induced iabetic wounds and high glucose-induced cell model, and the mechanism was systematically investigated by using RNA-seq technology.

## 2. Materials and Methods

### 2.1. Materials

Streptozotocin (STZ, SigmaS0130) and 4′,6-diamidino-2-phenylindole (DAPI) were purchased from Sigma-Aldrich. Huangbai liniment (batch number: 18010111) was kindly provided by Shandong Hanfang Pharmaceutical Co., Ltd (Chinese medicine character: Z10950097). Recombinant human epidermal growth factor derivative for external use (rhEGF) was purchased from Shenzhen Huashengyuan Gene Engineering Development Co., Ltd. The antibodies used in this research were as follows: Ki67 (ab92742), 8-OHdG (sc-66036), Nrf2 (ab137550), NQO1 (ab28947), cleaved caspase 3 (CST, 9664S), TGF-*β*1 (ab92486), goat anti-mouse IgG (H+L) (HRP Jackson, 115-035-003), and goat anti-rabbit IgG (H+L) (Jackson, 111-035-003). The malondialdehyde (MDA, A003) was purchased from Nanjing Jiancheng Bioengineering Institute. The ELISA kits of MMP9 (DY-0075) and 8-OHdG (DY-0223) were obtained from Deyi Testing Co., Ltd. Cell Counting Kit-8 (CCK-8) kits were obtained from the Dongren Chemical Technology Co., Ltd. The malondialdehyde ROS (S0033) and GSH (A119) were purchased from Beyotime Biotechnology and Nanjing Jiancheng Bioengineering Institute, respectively.

### 2.2. *In Vivo* Animal Study

Male Sprague-Dawley rats (170-200 g) were purchased from the Peking University Health Science Center Experimental Animal Center, Beijing, China ((certificate no. SCXK (Jing) 2009-0017)). All procedures were carried out according to the rules of the Committee on Animal Care and Use published by the Institute of Chinese Materia Medica, China Academy of Chinese Medical Sciences. Rats were housed in a 12-h light/dark cycle facility with a controlled temperature and kept with free access to water and food. Streptozotocin (STZ, 60 mg/kg, i.p.) in sodium citrate buffer (pH 4.5) was used to generate diabetic model according to previous reports [21]. The fasting glucose levels (FGLs) was evaluated 1 and 2 weeks following STZ injection. Rats with FGL more than 16.7 mmol were included for the following experiments. The control animals received the same volume of sodium citrate buffer (pH 4.5) instead. At two weeks after STZ induction, the rats were anesthetized with sodium pentobarbital (50 mg/kg, intraperitoneal injection, i.p.) and then subjected to full-thickness wounds. Two wounds with diameter of 2 cm on the back of rats were made. The rats were randomly allocated to different groups to receive either physiological saline, low dose of Huangbai liniment (STZ+HB-L, equal volume of HB+equal volume of physiological saline, 2 mL/day, topical), high dose of Huangbai liniment STZ+HB-H (original solution, 2 mL/day, topical), or rhEGF (4000 IU/10^∗^10cm^2^, topical) until study end. On the first three days after wound surgery, the wound was covered with double layers of sterile gauze to reduce external irritation and replaced daily. The wounds were photographed at 1, 6, 9, and 13 days after surgical wounds were made. The wound closure photos were analyzed using ImageJ by calculating the percentage of the wound area from the original wound area. After 13 days, skin tissues were collected, half of which were paraffin-embedded for histology, and the other half were used for western blotting, ELISA, and other related experiments.

### 2.3. *In Vitro* Cell Experiments

Human immortalized keratinocytes (HaCaT) purchased from National Infrastructure of Cell Line Resource was used for our *in vitro* experiment. Briefly, HaCaT cells were cultured with culture medium which contained DMEM (Gibco, USA) with D-glucose (1 g/L, LG), fetal bovine serum (10%v/v, FBS), and penicillin/streptomycin (100 *μ*g/mL) at 37°C, and stimulation of HaCaT cells with culture medium containing 4.5 g/L D-glucose DMEM (HG) for 48 hours was used for chronic hyperglycemic cell model. Povidone iodine and quercetin were purchased from Hubei Ketian Pharmaceutical Co., Ltd and National Institutes for Food and Drug Control, respectively. First, HaCaT cells were treated with various concentrations of HB, quercetin, and povidone iodine, respectively, for 48 hours to search the safe concentration. To evaluate the therapeutic effect, cells were treated with HG medium and drugs such as HB (1/50 and 1/5000, dilution), quercetin (1.5625 and 6.25 *μ*M), and povidone iodine (0.00001% and 0.001%, m/v) at the same time for 48 hours before detection. CCK-8 was used for detecting cell viability with a microplate reader (Molecular Devices, USA).

### 2.4. Measurement of Malondialdehyde, GSH, ROS, 8-OHdG, and MMP9

Malondialdehyde (MDA) was detected according to the manufacture instrument, using Visible Spectrophotometer 721G (Shanghai Precision Scientific Instruments Co., Ltd.). And GSH and ROS levels were also performed according to their instruction using a microplate reader (Molecular Devices, USA). The protein extract from the skin tissue was used for the detection of MMP9 and 8-OHdG using commercially available kits. Briefly, plates were immobilized with specific antibody (MMP9 or 8-OHdG) and incubated with protein samples, followed by a secondary antibody conjugated with horseradish peroxidase. The measurement was performed on Beijing DNM-9602G Enzyme Marker Analyzer.

### 2.5. Western Blotting

The skin tissue was harvested, and protein extraction was performed according to the instruction of Beyotime Protein Assay Kit (P0013, Nanjing, China). After separated on a 10% SDS-PAGE (P0014, China), the protein samples were transferred to a polyvinylidene fluoride membrane (Millipore, IPVH00010), which was blocked with bovine serum albumin (BSA). After that, the primary antibody specific for Nrf2 (ab137550) or NQO1 (ab28947) was added to the membranes and incubated for 24 h at 4°C. The second antibody goat anti-rabbit IgG (H+L) (Jackson, 111-035-003) was loaded, and the signal of the proteins was detected by scanning densitometry.

### 2.6. Histology and Immunofluorescence Staining

The paraffin tissue section slides with thickness of 5 *μ*m were used for histology and immunofluorescence staining. After deparaffinized, the slides were stained with hematoxylin and eosin (HE) and Masson's trichrome stain following their instructions, respectively. For immunofluorescence (IF) staining, the sections were blocked by 5% (m/v) BSA, after they were permeabilized with 0.5% Triton X-100. Primary antibody was loaded and kept at 4°C overnight. Then Alexa Fluor 488- and Alexa Fluor 647-conjugated secondary antibodies were loaded at room temperature for 1 h. The nuclei were stained with 4′,6-diamidino-2-phenylindole (DAPI). The images were visualized using LSM-880 confocal microscope (Carl Zeiss, Oberkochen, Germany). And the real-time collagen growth evaluation was conducted using two-photon microscope (Olympus FV1000) at 6 and 13 days after wound generation.

### 2.7. Investigation and Analysis of Differentially Expressed Genes Identified by RNA-Seq Technology

The skin tissue was harvested, and the RNA was separated by using Total TRIzol Reagent (Cat#15596-018, Life Technologies, USA) following its instructions. RNA integrity was quantified with the RNA Nano 6000 Assay Kit using Bioanalyzer 2100 system (Agilent Technologies, CA, USA). The RNA (1 *μ*g) of each sample was used for the following library construction. The RNA sequencing was performed according to the previous research [15]. Briefly, NEBNext® Ultra™ RNA Library Prep Kit was applied to produce the sequencing libraries for Illumina® (NEB, USA). Firstly, purification of mRNA was carried out by using poly-T oligo-grafted magnetic beads before RNA fragmentation was generated. After that, the first strand cDNA was synthesized by using M-MuLV Reverse Transcriptase(RNase H-) and random hexamer primer. And the second strand cDNA was synthesized with RNase H and DNA polymerase I. The enrichment of cDNA template by PCR was carried out after knot of the adapter and adenylation at the 3′ end in the DNA fragment. After purification of PCR results, the library quality was evaluated, and the clustering of samples was performed on TruSeq PE150 Cluster Kit v3-cBot-HS (Illumia) with a cBot Cluster Generation System. Finally, the sequence of the library was carried out by using an Illumina HiSeq 4000 platform with the generation of 150 bp paired-end reads. Novogene Bioinformatics Technology Co., Ltd (Beijing, China) helped to do the sequencing experiment.

Rat reference genome (ensemble release 91) was applied for the transcript assembly and quantification. The numbers of reads were calculated by mapping to every gene with HTseq v0.6.1. Gene expression was obtained by evaluating the fragment number of each kilobase of transcript sequence normalized to every million base pairs which were sequenced and expressed as FPKM. Differential expression of genes was calculated with EdgeR software. The raw data related to HB-mediated protection against STZ-induced diabetic wound was uploaded into https://www.ncbi.nlm.nih.gov/sra/PRJNA532974 (SRP193129). The differentially expressed genes identified by RNA-seq were analyzed through ClueGO. And the network of “components-drug targets-differentially expressed genes” was constructed with Cytoscape v3.4.0. [22]. Briefly, the drug targets of HB liniment were obtained through BATMAN-TCM, and the downstream differentially expressed genes of Nrf2 and Nfe2l2 were associated with the drug targets by STRING to screen out related drug targets, and the network was constructed through Cytoscape v3.4.0.

### 2.8. Statistical Analysis

The data was analyzed using one-way ANOVA with Tukey post hoc test through SPSS software, and the data was presented as means ± SD (standard deviations). ^#^*P* < 0.05 compared with the Con or LG group, ^∗^*P* < 0.05 compared with the STZ or HG group was considered as statistical significance.

## 3. Results

### 3.1. HB Liniment Accelerated Wound Closure in STZ-Induced Diabetic Rats

**Figure 1:**
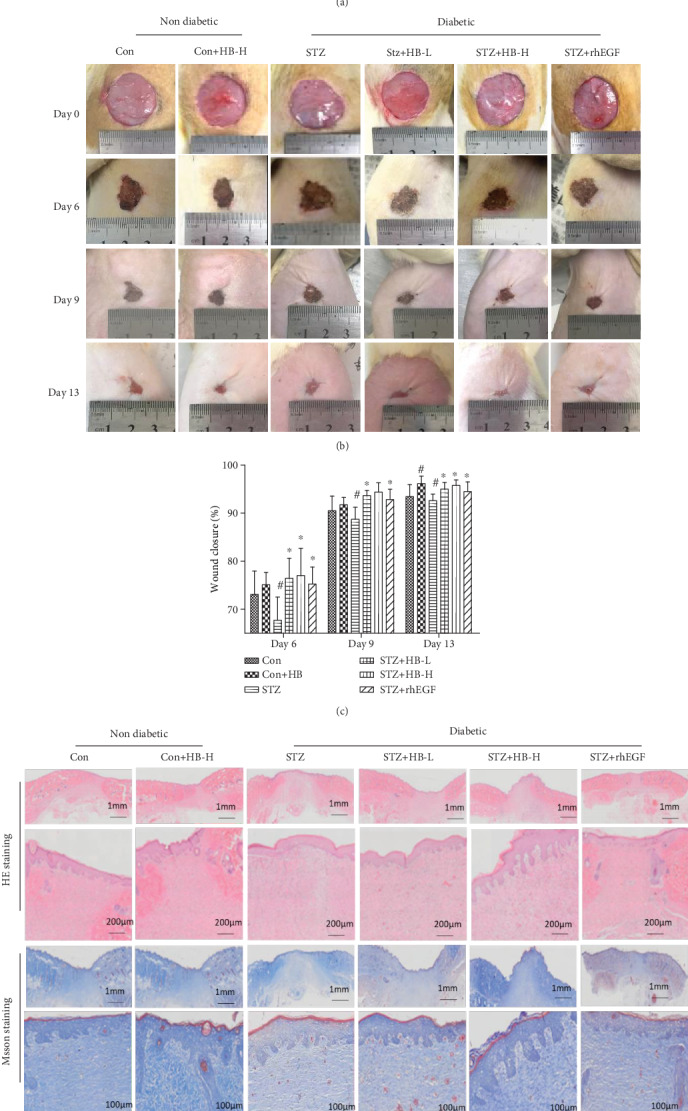
HB treatment accelerated wound closure in STZ-induced diabetic model. (a) Schematic showing diabetes induction and wound surgery generation treated with HB liniment for 13 days. (b) Representative photos of wounds healing in different time points with different treatments. (c) Wound closure analyzed by ImageJ (*n* = 10). Data were expressed as mean ± SD, and significance was expressed as ^#^*P* < 0.05 vs Con and ^∗^*P* < 0.05 vs STZ. (d) Hematoxylin and eosin staining and Masson staining of the wound healing tissues at day 13 after wound surgery.

The effect of HB liniment on diabetic wound healing was investigated through the evaluation of the wound closure and histology staining. As indicated in Figures 1(b) and 1(c), the wound closure in the STZ group was slower than those of the control group. Both low- and high-dose HB liniments increased the wound closure to the level of the STZ+rhEGF group in STZ-induced diabetic wounds, but it cannot promote wound healing of nondiabetic rats until at 13 days after operation. Additionally, HB liniment and rhEGF treated rats demonstrated a stronger staining than that of the STZ group as indicated by HE staining and Masson staining (Figure 1(d)), indicating improved ECM synthesis and collagen production by HB and rhEGF treatments. These results demonstrated that HB treatment promoted wound healing in STZ-induced diabetic rats.

### 3.2. The Mechanism of HB-Mediated Wound Healing Was Systematically Investigated by RNA-Seq Technology

**Figure 2:**
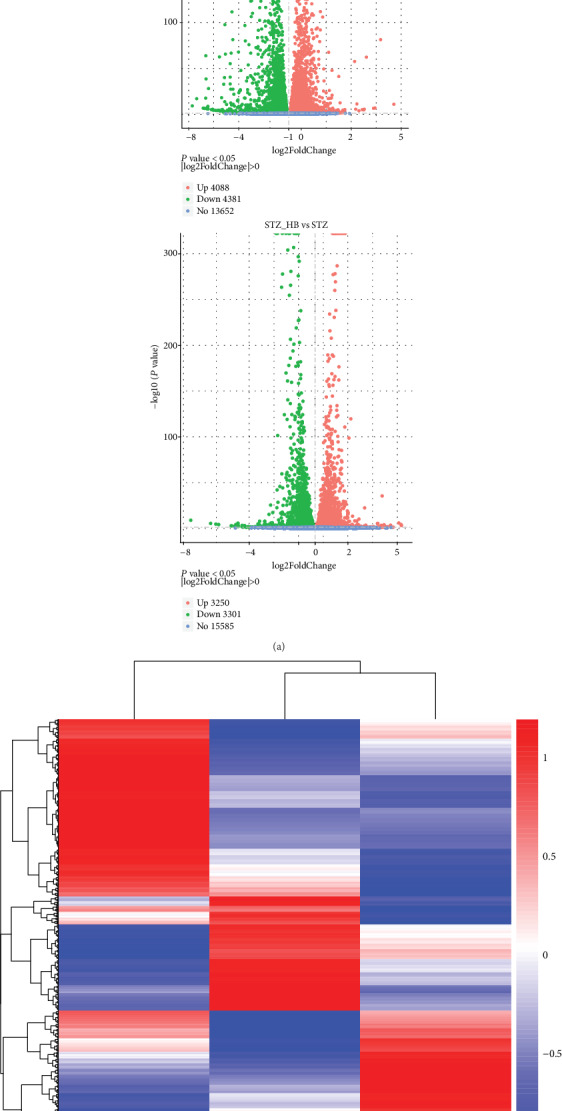
Gene expression profiling of HB-mediated wound healing in STZ-induced diabetes. (a) Differentially expressed genes (DEs) in the STZ group compared with the Con group (nondiabetic rats) and HB-treated group compared with the STZ group; upregulated DEs were shown as red dots, and downregulated DEs shown green with no significant genes as blue dots; (b) hierarchical clustering of differentially expressed genes presented as a heat map of different groups.

**Figure 3:**
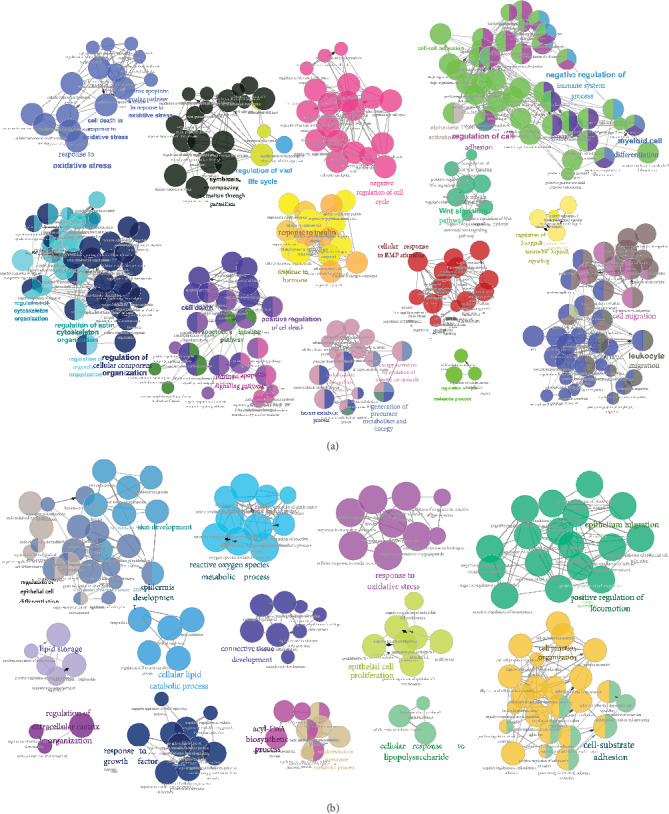
Enrichments of differentially expressed genes in HB-mediated wound healing by using ClueGo within Cytoscape software. (a) Enriched biological processes of DEs between the STZ group and the Con group; (b) enriched biological processes of DEs between the STZ+HB group and the STZ group.

To reveal the underlying mechanism, RNA-seq technology was applied to systematically investigate the gene expression after various treatments. In contrast to the control, there were 4088 upregulated and 4381 downregulated genes in the STZ group. HB liniment significantly upregulated 3250 genes and downregulated 3301 genes when compared to the STZ group (Figure 2(a)). And the gene pattern of the STZ+HB group was more similar to the control group rather than the STZ group (Figure 2(b)). Additionally, the DEs between STZ and control were enriched into overrepresented GO terms including “response to oxidative stress,” “cell death response to oxidative stress,” “regulation of cytoskeleton organization,” “cell death,” “apoptotic signaling pathway,” and so on (Figure 3,). Whereas, skin growth-related GO terms such as “skin development,” “regulation of extracellular matrix organization,” “connective tissue development,” and “epithelial cell proliferation” were observed after HB treatment, indicating HB treatment improved wound healing in STZ-induced diabetic model. Importantly, oxidative stress-related GO terms such as “reactive oxygen species metabolic process” and “response to oxidative stress” and cell migration-related GO terms such as “positive regulation of locomotion” and “epithelium migration” were also found in the STZ+HB group. These data indicated oxidative stress and extracellular matrix secretion may be important factors in HB-mediated wound healing.

### 3.3. HB Liniment Inhibited Oxidative Damage and Apoptosis in STZ-Induced Diabetic Rats

**Figure 4:**
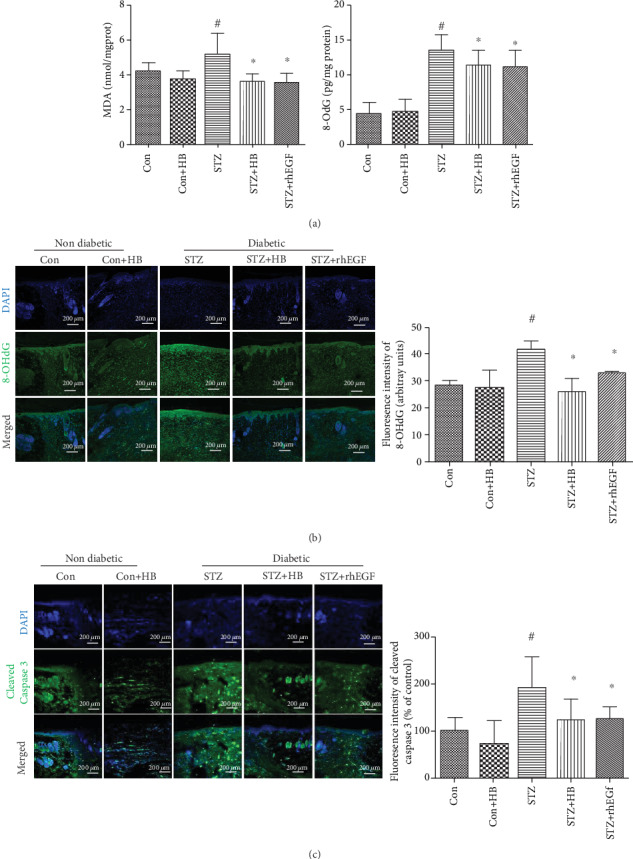
HB treatment inhibited oxidative damage and reduced cell apoptosis in diabetic wound model. (a) The level of MDA and 8-OHdG (*n* = 6); (b) immunofluorescence staining of 8-OHdG (green) and its quantitative results (*n* = 3 − 5 animals per group); scale bar: 200 *μ*m, nucleus (blue); (c) immunofluorescence staining of cleaved caspase 3 (green) and its quantitative results (*n* = 3 − 5 animals per group); scale bar: 200 *μ*m, nucleus (blue); The data were expressed as mean ± SD and significance was expressed as ^#^*P* < 0.05 vs Con and ^∗^*P* < 0.05 vs STZ.

To verify the antioxidant capacity of HB in diabetic rats, the amount of MDA and 8-OHdG was measured accordingly. As indicated by Figure 4(a), increased oxidative products such as MDA and 8-OHdG were observed in the STZ group. However, the increased MDA and 8-OHdG in the STZ group was reduced by HB and rhEGF treatment. The immunofluorescence staining also confirmed the lower staining of 8-OHdG in the STZ+HB group than that of the STZ group. However, no significance was found between the control group and the Con+HB group (Figure 4(b)). Moreover, cell apoptosis was also repressed by HB liniment and rhEGF as indicated by the less staining of cleaved caspase 3 than that of the STZ group (Figure 4(c)). Taken together, oxidative damage and cell apoptosis were significantly inhibited by HB liniment and rhEGF.

### 3.4. HB Liniment Activated Nrf2 and Its Downstream Genes in STZ-Induced Diabetic Rats

**Figure 5:**
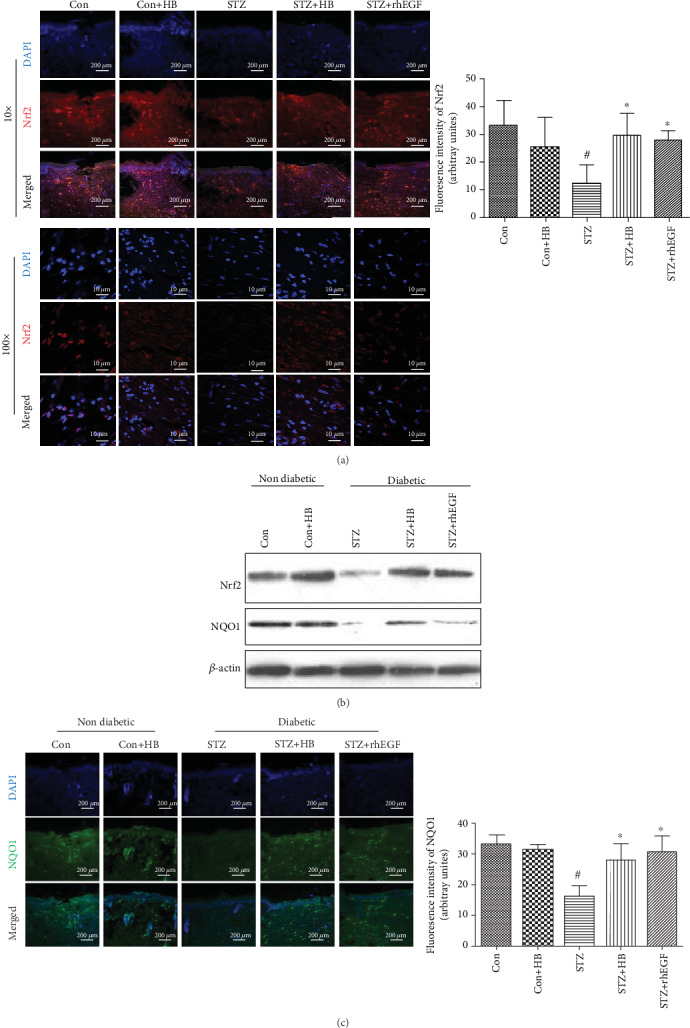
HB treatment-activated Nrf2 and its downstream NQO1 in STZ-induced wound model. (a) immunofluorescence staining of Nrf2 (red) and its quantitative results (*n* = 3 − 5 animals per group); nucleus (blue); (b) the representative western blotting results of Nrf2 and NQO1; (c) immunofluorescence staining of cleaved caspase 3 (green) and its quantitative results (*n* = 3 − 5 animals per group); scale bar: 200 *μ*m, nucleus (blue); the data were expressed as mean ± SD, and significance was expressed as ^#^*P* < 0.05 vs Con and ^∗^*P* < 0.05 vs STZ.

Since nuclear factor erythroid-derived 2-like 2 (Nrf2) plays as central regulator in the maintenance of redox homeostasis by recognizing antioxidant response element and activates antioxidant genes, the Nrf2 level and its downstream NQO1 were determined accordingly. As indicated in Figures 5(a) and 5(b), STZ group had an obvious reduction in Nrf2 expression, which was significantly reversed by HB liniment and rhEGF treatment. Importantly, the nuclear staining of Nrf2 in STZ-induced diabetic rats was obviously weak whereas it was enhanced by HB liniment. Additionally, the decreased NQO1 in the STZ group was also enhanced by HB liniment and rhEGF (Figures 5(b) and 5(c)).

**Figure 6:**
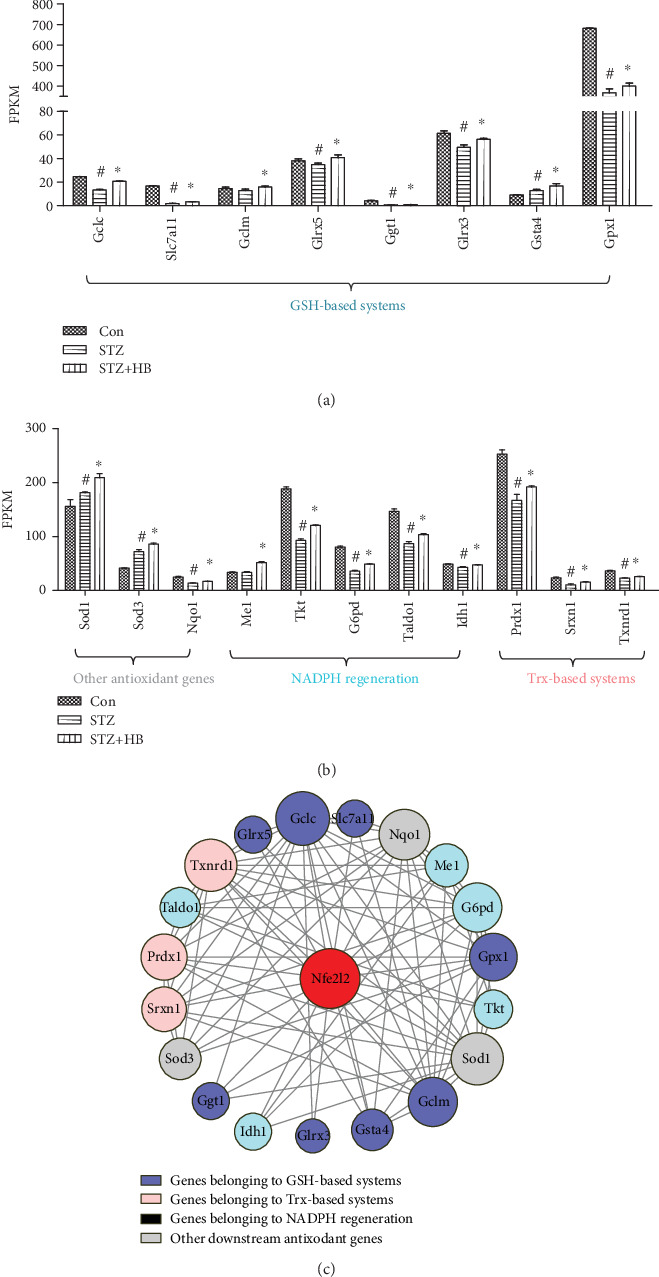
HB treatment activated the downstream genes of Nrf2 in diabetic wound model. (a) The downstream genes of Nrf2 involved in GSH-based antioxidant systems (*n* = 3); (b) The downstream genes of Nrf2 involved in Trx-based antioxidant systems, GAPDH generation and other antioxidant processes (*n* = 3); (c) The network of Nfe2l2 and its downstream DEs constructed by Cytoscape software. The data were expressed as mean ± SD, and significance was expressed as ^#^*P* < 0.05 vs Con and ^∗^*P* < 0.05 vs STZ.

To further verify the activity of Nrf2, its downstream DE genes which were involved in resisting oxidative stress were listed in Figure 6. A large number of Nrf2 downstream antioxidant genes such as Gclc, Slc7a11, Glrx5, Ggt1, Glrx3, Gsta4, Gpx1, Prdx1, Srxn1, Txnrd1, Tkt, G6pd, Taldo1, Idh1, Sod1, Sod3, and NQO1 were significantly repressed in the STZ group. Importantly, GSH-based antioxidant genes including Gclc, Gclm, Slc7a11, Glrx5, Ggt1, Glrx3, Gsta4, and Gpx1; Trx-based antioxidant genes including Prdx1, Srxn1, and Txnrd1; NADPH generation-related antioxidant genes including Me1, Tkt, G6pd, Taldo1, and Idh1, as well as other antioxidant genes including Sod1, Sod3, and NQO1 were all activated by HB liniment. Moreover, a close relationship of Nrf2 and its downstream genes was observed (Figure 6(c)). Thus, HB liniment treatment enhanced Nrf2 and its downstream antioxidant genes to attenuated oxidative damage.

### 3.5. Identification of Critical Components in HB That Affected Nfe2l2 and Its Downstream Genes by Network Pharmacology Analysis

**Figure 7:**
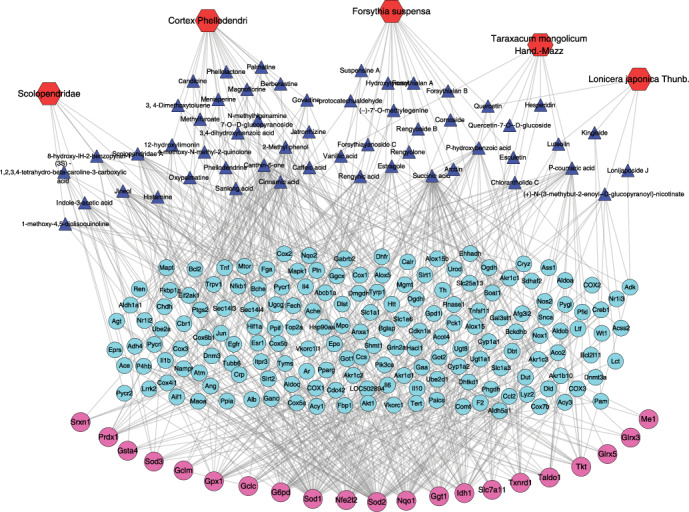
The components of HB liniment which affected Nfe2l2 and its downstream differentially expressed genes (DEs) were identified through Network pharmacology analysis. Specifically, 54 components in HB liniment were identified with 21 components from Cortex Phellodendri, 18 components from Forsythia suspensa, 9 components from Taraxacum mongolicum Hand.-Mazz, 5 from Lonicera japonica Thunb., and 7 from Scolopendridae. Some components generated from multiple Chinese medicine. Red hexagons indicated the Chinese medicine, blue triangles indicated the components of HB, blue dots indicated drug targets of components from HB liniment, and red dots indicated Nfe2l2 and its downstream DEs.

To identify the components which affected Nfe2l2 and its downstream differentially expressed genes (DEs), drug targets of HB liniment were associated with Nfe2l2 and its downstream DEs and a network of components-drug targets-DE genes was constructed. As indicated by Figure 7, 54 components in HB liniment were identified through 169 drug targets by associating with Nfe2l2 and its downstream DEs. Specifically, 21 components from Cortex Phellodendri including Cinnamic acid, caffeic acid, Jatrorrhizine, 3,4-dihydroxybenzoic acid, sanleng acid, and 16 other components while 18 components from Forsythia suspensa including suspensine A, hydroxytyrosol, vanillic acid, Succinic acid, and other 14 components were screened out. Additionally, there were 9 components from Taraxacum mongolicum Hand.-Mazz, 5 from Lonicera japonica Thunb, and 7 from Scolopendridae were identified to have an effect on the differentially expressed oxidative genes. Among these identified components, there were several components originated from multiple drugs. For example, Quercetin generated from both Forsythia suspense, Lonicera japonica Thunb, and Taraxacusm mongolicum Hand.-Mazz.

### 3.6. HB Liniment Modulated the Expression of MMP9 and TGF-*β*1 and Improved Collagen Growth

**Figure 8:**
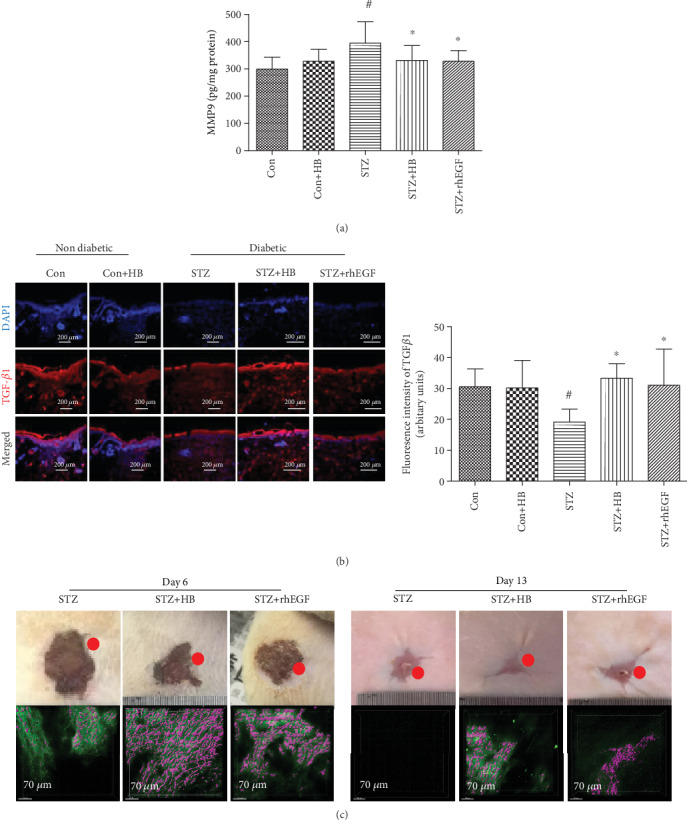
HB treatment enhanced TGF-*β*1 expression, decreased MMP9 production, and facilitated collagen growth. (a) immunofluorescence staining of TGF-*β*1 (red) and its quantitative results (*n* = 3 − 5 animals per group); scale bar: 200 *μ*m, nucleus (blue); (b) MMP9 level (*n* = 6); (c) New collagen growth observed by using two-photon microscope at days 6 and 13 after wound surgery. The green indicated the background, and the purple indicated the new collagen tissue at the front of the wound healing. The data were expressed as mean ± SD, and significance was expressed as ^#^*P* < 0.05 vs Con and ^∗^*P* < 0.05 vs STZ.

To further reveal the mechanism of HB, TGF-*β*1, MMP9 level, and the collagen growth were evaluated accordingly. As indicated by Figure 8, the decreased TGF-*β*1 and elevated MMP9 in the STZ group were significantly reversed by HB liniment and rhEGF. The neo-collagen growth at the front of the wound healing was increased by both HB liniment and rhEGF in contrast to the STZ group. These results showed HB liniment and rhEGF enhanced TGF-*β*1, reduced MMP9, and improved collagen growth.

### 3.7. HB Liniment and Quercetin Promoted Cell Proliferation and Decreases Oxidative Damage in HG-Induced Model

**Figure 9:**
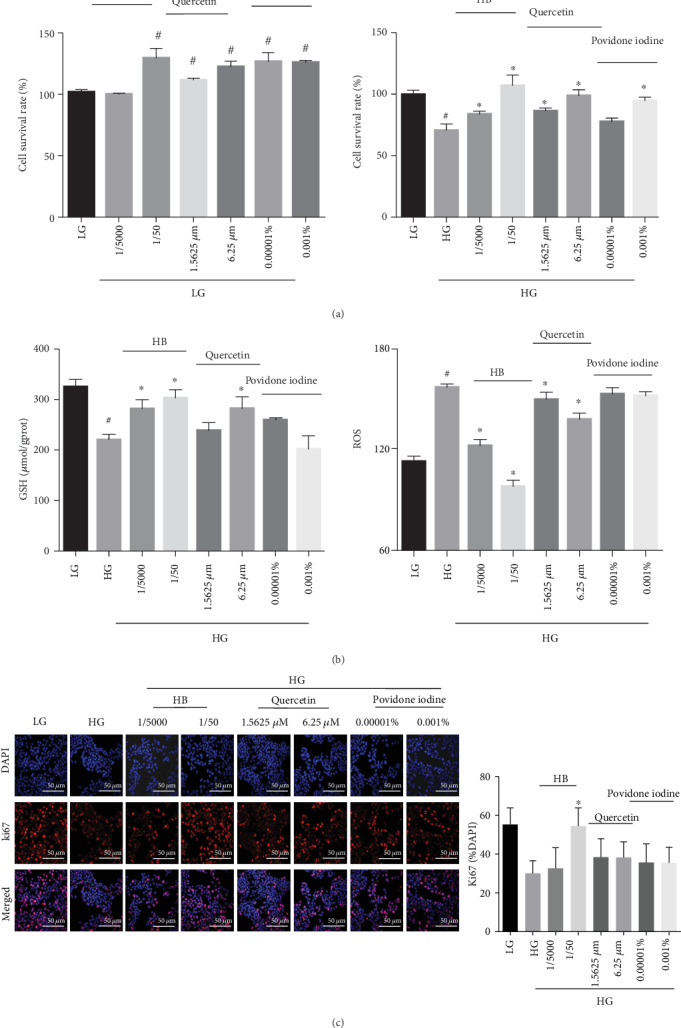
HB facilitated cell proliferation and reduced oxidative damage in HaCaT cells. (a) Cell viability in LG and HG (*n* = 6); (b) the levels of GSH (*n* = 3) and ROS (*n* = 6); (c) immunofluorescence staining of Ki67 (red) and its quantitative result (*n* = 3 − 5 per group), nucleus (blue), scale bar: 50 *μ*m; the data were expressed as mean ± SD, and significance was expressed as ^#^*P* < 0.05 vs LG and ^∗^*P* < 0.05 vs HG.

To further confirm the mechanism, the effect of HB and quercetin (one of its components), which was reported to have an effect on diabetic wounds, was tested using HaCaT cells. As indicated by Figure 9, HB and quercetin did not inhibit cell viability but enhanced cell proliferation in low-glucose medium. HG stimulation caused a remarkably decrease in cell viability, whereas this was ameliorated by HB, quercetin, and povidone iodine. To confirm the effect of HB on oxidative stress, the levels of ROS and GSH were measured accordingly. HG stimulation led to a significant decrease in GSH level, which was dramatically increased by HB and quercetin. Additionally, the increased ROS level induced by HG was also significantly reduced by HB and quercetin but not by povidone iodine. The positive rate of Ki67 (a cell proliferation marker) was decreased by HG stimulation, which was significantly increased by HB, but not by quercetin or povidone iodine (Figure 9(c)). These data indicated that HB had a good effect in attenuating oxidative damage and promoting cell proliferation.

## 4. Discussion

**Figure 10:**
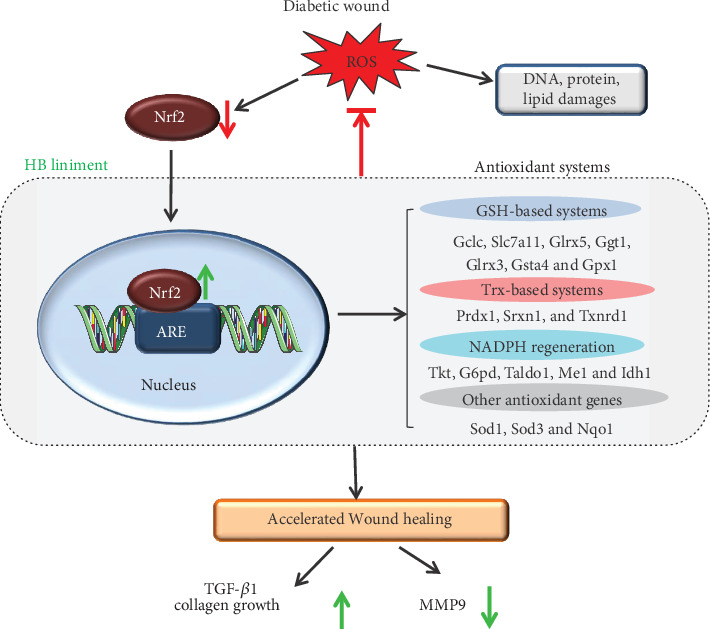
Schematic diagram illustrating of the potential mechanism of HB-mediated diabetic wound healing. Reactive oxygen species in diabetic wound caused DNA double strands breakage, lipid peroxide, protein denaturation, and impaired diabetic wound healing. Specifically, Nrf2 and its downstream antioxidant genes were significantly decreased, and wound healing was impaired. In contrast, HB liniment markedly increased Nrf2 level and its downstream genes involved in GSH- and Trx-based systems, NADPH generation, and other antioxidant systems; this led to enhanced antioxidant capacity and decreased oxidative and apoptosis damage. Additionally, these also increased TGF-*β*1 and collagen growth while decreased MMP9, which accelerated wound closure in HB-mediated wound healing.

Diabetic wounds cause high mortality and disability in a large number of diabetic patients, whereas there is a limited effective therapy. In this study, the effect of Huangbai (HB) liniment on STZ-induced diabetic wounds was systematically investigated, and the mechanism was revealed. HB liniment significantly accelerated the wound closure and enhanced the generation of extracellular matrix in STZ-induced diabetic rats. By using RNA-seq technology analysis, oxidative stress was identified as a vital process in HB-mediated wound healing. Importantly, the activation of Nrf2 and its downstream genes involved in glutathione system, thioredoxin system, and NADPH generation as well as other antioxidant genes were observed after HB treatment. The activated Nrf2 and its downstream genes developed a stable antioxidant network, contributing to the reduction of oxidative damage and apoptosis (Figure 10). Moreover, HB liniment enhanced TGF-*β*1, reduced MMP9 level, and promoted collagen growth, thereby accelerating wound healing in diabetic conditions. The *in vitro* experiment demonstrated the good effect of HB in inhibiting oxidative damage and enhancing cell proliferation. By associating drug targets of HB liniment with Nrf2 and its downstream differentially expressed genes, 54 components were screened out, and the majority of the components was from Cortex Phellodendri and Forsythia suspensa.

Growing evidence has accumulated that redox homeostasis serves a significant role in modulating diabetic wound healing [23]. Overproduced ROS in diabetes damages macromolecules such as lipids, proteins, and DNA double strands, thus results in impaired wound healing and apoptosis. In our study, oxidative stress was identified as a vital process in HB-mediated wound healing (Figure 3), which was subsequently confirmed by decreased high DNA damage (8-OHdG), peroxide products (MDA), and reduced apoptosis in STZ rats. As a central regulator of cellular redox status, Nrf2 is responsible for modulating transcription of cytoprotective and antioxidant genes, which affects the ability of wounds to heal in diabetics [24]. Activation of Nrf2 by its activator or inhibiting its repressor Keap1 alleviated ROS generation, decreased oxidative damage, and accelerated wound closure in diabetes [25, 26]. On the contrary, Nrf2^−/−^ mice was characterized by delayed wound healing [25], indicating the vital role of Nrf2 in facilitating wound closure in diabetes. As consistently demonstrated in our study, hindered Nrf2 activity in diabetes failed to modulate redox homeostasis and resulted in impaired wound closure, whereas the Nrf2 signaling restored by HB treatment inhibited oxidative damage and promoted wound healing. In addition, our results demonstrated the dysfunction of its target genes involved in GSH-based, Trx-based antioxidant systems, as well as NADPH-generating process and other antioxidant genes such as NQO1, Sod1, and Sod3 were also rescued by HB treatment in STZ-induced diabetic wound (Figure 6). Nrf2 activation enhanced expression of Gclc and Gclm which determined the GSH biosynthesis, Gpx1 which helped to decompose H_2_O_2_ and produce GSSG, and Ggt1 which initiated extracellular glutathione (GSH) breakdown. The elevated levels of GSH-based antioxidant genes maintained GSH homeostasis and ensured that its antioxidant effect was realized [27]. Moreover, Nrf2 activation upregulated gene expression of Txnrd1, Srxn1, and Prdx1, which allowed for the reduction of oxidized thiols in proteins [28]. As a vital reducing resource, NADPH was required for the GSH and Trx regeneration to eliminate excess ROS [29]. The elevated gene expression of Me1, Tkt, Idh1, and Taldo1 by Nrf2 demonstrated the improved function of NADPH generation and maintained GSH- and Trx- based antioxidant function. Thus, the activated Nrf2, together with its downstream genes involved in GSH-based, Trx-based antioxidant systems, as well as NADPH generating process and other antioxidant genes, eliminated ROS, decreased oxidative damage and apoptosis, and accelerated wound healing in diabetes.

The compounds in HB liniment that affected Nle2l2 and its downstream genes were identified accordingly. Specifically, 54 components from HB liniment were identified to have an effect on Nle2l2 and its downstream antioxidant genes. Significantly, among the 21 identified components from Cortex Phellodendri, Cinnamic acid [30], caffeic acid [31], Magnoflorine [32, 33], Jatrorrhizine [34], phellodendrine, and 3,4-dihydroxybenzoic acid have proven to reduce oxidative stress. The extract of Forsythia suspensa exhibited a significant antihyperglycemic and antihyperlipidemic effect in the treatment for diabetes by modulating oxidative stress and insulin secretion [35]. The 18 compounds from Forsythia suspensa, including quercetin [36], vanillic acid [37], Arctiin [38], hydroxytyrosol [39] and Protocatechualdehyde [40], ameliorated oxidative damage. Additionally, Taraxacum mongolicum Hand.-Mazz [41]. and its identified components such as p-Coumaric acid and Esculetin [42, 43], as well as Lonicera japonica Thunb., and its identified components such as Luteolin had a beneficial antioxidant effect by removing ROS [44, 45]. Moreover, no studies have shown that Scolopendridae were associated with diabetes, although they have demonstrated to exhibit good antioxidant capacity [46]. In this study, the antioxidant property of HB liniment was observed in the treatment of STZ-induced diabetic wound by using the combination of Cortex Phellodendri, Forsythia suspensa, Lonicera japonica Thunb., Taraxacum mongolicum Hand.-Mazz, and Scolopendridae. In addition, quercetin, which came from three of the five compositions of HB liniment, demonstrated multiple effects in facilitating wound healing [47]. Researches showed it not only has good antibacterial action, similar to other standard drugs (e.g., povidone iodine) [48], but also decreased inflammation, inhibited oxidative damage, and enhanced secretion of VEGF and TGF-*β*1, modulated integrin expressions to reduce fibrosis during wound healing [47, 49]. In this study, the effect of HB, quercetin, and povidone iodine on inhibiting oxidative damage was compared in HG-induced HaCaT cell model. It demonstrated that both HB and quercetin had a good effect in inhibiting oxidative damage rather than povidone iodine (Figure 9). Thus, four herbs and Scolopendridae in HB liniment shared some same components, and the 54 identified compounds had a synergistically inhibitive effect on oxidative stress through modulating Nrf2 and its downstream antioxidant genes.

Along with the enhanced antioxidant capacity by HB liniment, an increase in transforming growth factor-*β*1 (TGF-*β*1) and reduction in matrix metalloproteinase (MMP) contributed to the increased extracellular matrix growth and enhanced wound healing in diabetes. Elevated levels of ROS hinder tissue growth, especially for collagen production and halt wound closure [12], and the interference of ECM production is highlighted through the disruption of TGF-*β*1 signaling [10, 11]. Besides, high ROS level affects ECM remodeling through regulating MMPs expression, and ROS production is required for MMPs synthesis [13]. These were consistent with our results that serious oxidative damage in diabetic rats was associated with low ECM synthesis and collagen generation. Delayed wound healing in diabetes is usually characterized with low TGF-*β*1 and high MMPs in skin tissue of patients with diabetic foot ulcer [50–52]. TGF-*β*1 signaling plays a critical role as the regulator in a series of biological processes of skin regeneration and wound healing, such as angiogenesis and reepithelialization. [53]. MMP9 is responsible for eliminating damaged extracellular matrix and allows for skin tissue remodeling [54]. An overproduction of MMP9 in diabetes causes excessive ECM degradation and results in delayed wound healing [54], but inhibition or knockout of MMP9 promotes diabetic wound healing [55]. This indicates a critical role of MMP9 in treating diabetic ulcers. Consistent with these research findings, decreased TGF-*β*1 and elevated MMP9 were observed in our diabetic wound model, and these were significantly alleviated by HB treatment as indicated by significantly higher TGF-*β*1 and reduced MMP9 level. Recent research indicates that Nrf2 activation could promote TGF-*β*1 generation and inhibit the overproduction of MMP9 in diabetic wound [25] Our results demonstrated that elevated TGF-*β*1 and decreased MMP9 were associated with the activation of Nrf2 in HB-mediated wound healing.

Taken together, our findings demonstrated activation of Nrf2 by HB liniment accelerated diabetic wound healing. This study provided experiment evidence for topical HB liniment treatment for diabetic wounds and highlighting the effect of activating Nrf2 in diabetic wound healing. However, we only focused on the effect of HB on oxidative damage, whether it had an effect on inflammation, angiogenesis and antibiosis remained further investigation. In addition, the components in HB liniment which really played a role in activating Nrf2 need to be clarified in the future study.

## 5. Conclusions

In summary, an obvious protective effect of HB treatment against diabetic wound healing was observed, and reduction in oxidative stress may play a vital role in HB-mediated wound healing. RNA-seq analysis and further experiment suggested that the activation of Nrf2 and its downstream antioxidant genes reduced apoptosis and oxidative damage, thereby improving HB-mediated wound healing. And 54 compounds in HB liniment were identified to have an effect on Nfe2l2 and its downstream genes, which had a synergistically inhibitive effect on oxidative stress. In addition, HB liniment, increased TGF-*β*1 level, and reduced MMP9, thereby contributing to the acceleration of diabetic wound healing. Our findings offered experimental evidence of HB liniment in the treatment of diabetic wounds and also improved our understanding of diabetic wound healing, supporting development of Nrf2-based therapy in the treatment of diabetic ulcer.

## Data Availability

The data about the RNA-seq analysis was uploaded into NCBI https://www.ncbi.nlm.nih.gov/sra/PRJNA532974 (SRP193129) which can be downloaded. As for the other data used to support the findings of this study are available from the corresponding author upon request.

## Supplementary Materials

Supplementary Materials Table S1. The RNA-seq data of nondiabetic wound (Con), STZ-induced diabetic wound with or without HB treatment. The gene expressions of different ss were expressed as FPKM and the fold change and false discovery rate (FDR).
